# Type 2 diabetes, obesity, and risk of amyotrophic lateral sclerosis: A population‐based cohort study

**DOI:** 10.1002/brb3.3007

**Published:** 2023-04-19

**Authors:** Nils Skajaa, Emil Bjerregaard Riahi, Szimonetta Komjáthiné Szépligeti, Erzsébet Horváth‐Puhó, Trine Toft Sørensen, Victor W. Henderson, Henrik Toft Sørensen

**Affiliations:** ^1^ Department of Clinical Epidemiology Aarhus University Hospital and Aarhus University Aarhus Denmark; ^2^ Institute of Public Health University of Copenhagen Copenhagen Denmark; ^3^ Department of Epidemiology and Population Health Stanford University Stanford California; ^4^ Department of Neurology and Neurological Sciences Stanford University Stanford California; ^5^ Clinical Excellence Research Center Stanford University Stanford California

**Keywords:** amyotrophic lateral sclerosis, cohort study, diabetes, obesity, population‐based

## Abstract

**Background:**

Type 2 diabetes and obesity may be inversely associated with amyotrophic lateral sclerosis (ALS), but the evidence is controversial.

**Methods:**

Using Danish, nationwide registries (1980‐2016), we identified patients with a diagnosis of type 2 diabetes (*N* = 295,653) and patients with a diagnosis of obesity (*N* = 312,108). Patients were matched (1:3) to persons from the general population on birth year and sex. We computed incidence rates and Cox regression derived hazard ratios (HRs) of a diagnosis of ALS. In multivariable analyses, HRs were controlled for sex, birth year, calendar year, and comorbidities.

**Results:**

We observed 168 incident cases of ALS (0.7 [95% confidence interval (CI): 0.6–0.8] per 10,000 person‐years) among patients with type 2 diabetes and 859 incident cases of ALS (0.9 [95% CI: 0.9–1.0] per 10,000 person‐years) among matched comparators. The adjusted HR was 0.87 (95% CI: 0.72–1.04). The association was present among men (adjusted HR: 0.78 [95% CI: 0.62–0.99]) but not women (adjusted HR: 1.03 [95% CI: 0.78–1.37]), and among those aged ≥60 years (adjusted HR: 0.75 [95% CI: 0.59–0.96]) but not younger. We observed 111 ALS events (0.4 [95% CI: 0.4–0.5] per 10,000 person‐years) among obesity patients and 431 ALS events (0.5 [95% CI: 0.5–0.6] per 10,000 person‐years) among comparators. The adjusted HR was 0.88 (95% CI: 0.70–1.11).

**Conclusions:**

Diagnoses of type 2 diabetes and obesity were associated with a reduced rate of ALS compared with general population comparators, particularly among men and patients aged 60 years or above. However, absolute rate differences were small.

## Graphical abstract

Type 2 diabetes and obesity may be inversely associated with amyotrophic lateral sclerosis (ALS), but the evidence is controversial. In a nationwide cohort study, diagnoses of type 2 diabetes and obesity were associated with a reduced rate of ALS compared with general population comparators, particularly among men and patients aged 60 years or above.

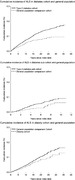

## 1 INTRODUCTION

Amyotrophic lateral sclerosis (ALS) is a neurodegenerative disease with insidious onset and characterized by progressive loss of motor neurons in the brain and spinal cord, which usually culminates in death (van Es et al., 2017). The incidence is higher in men than in women, and the prognosis is poor. The median survival from symptom onset has been reported to be 32 months (del Aguila et al., 2003) and respiratory paralysis is a common cause of death (Brown & Al‐Chalabi, 2017).

The pathogenesis of ALS remains poorly understood. Apart from male sex, older age, and familial history, few known risk factors for ALS exist (Ingre et al., 2015). Growing evidence associates defective energy metabolism with ALS, including weight loss, hypermetabolism, and hyperlipidemia (Seelen et al., 2014), and these alterations may occur before clinical manifestations of ALS (Seelen et al., 2014). Prior medical conditions, particularly those of cardiometabolic origin, may also impact the risk of ALS (Hollinger et al., 2016; Mitchell et al., 2015; Seelen et al., 2014). For example, several reports, mostly case‐control analyses, suggested that diabetes and obesity, conditions both associated with defective energy metabolism (Seelen et al., 2014), protect against ALS (D'Ovidio et al., 2018; Hollinger et al., 2016; Kioumourtzoglou et al., 2015; Mariosa et al., 2015; Mitchell et al., 2015; O'Reilly et al., 2013; Seelen et al., 2014; Sun et al., 2015; Tsai et al., 2019). For example, in a Danish case‐control study, Kioumourtzoglou et al. (2015) found a large protective effect of diabetes (odds ratio: 0.61 [95% confidence interval (CI): 0.46−0.80]). In a Swedish case‐control study, Mariosa et al. (2015) found a similar effect size (odds ratio: 0.79 [95% CI: 0.68−0.91]). Three cohort studies, two from Taiwan (Sun et al., 2015; Tsai et al., 2019) and one from Italy (D'Ovidio et al., 2018), showed divergent results (hazard ratio point estimates ranging from 0.30 to 1.35). A systematic review concluded that the evidence of an association between diabetes and ALS risk is limited, calling for further evidence from cohort studies (Lekoubou et al., 2014).

We examined the association between type 2 diabetes and ALS and that between obesity and ALS using Danish registry data. We hypothesized that hospital‐based diagnoses of type 2 diabetes and obesity were inversely associated with incident ALS.

## 2 METHODS

### 2.1 Design and setting

We conducted a population‐based matched cohort study in Denmark based on nationwide administrative registry data between January 1, 1980 and December 31, 2016. Denmark has a tax‐supported health care system, ensuring equal access to general practice and hospital‐based care (Schmidt et al., 2019). A unique personal identification number, assigned to all residents at birth or upon immigration, permits unambiguous, individual‐level linkage across data sources (Schmidt et al., 2014).

### 2.2 Type 2 diabetes and obesity cohorts

We used the Danish National Patient Registry (DNPR) (Schmidt et al., 2015) to identify a cohort of patients with a first‐time hospital‐based diagnosis of type 2 diabetes mellitus and another cohort of patients with a first‐time hospital‐based diagnosis of obesity. For both cohorts, we searched for both primary and secondary and inpatient and outpatient clinic diagnoses between January 1, 1980 and December 31, 2016. We excluded patients with a prior diagnosis of ALS or other motor neuron diseases (122 diabetes patients and 71 obesity patients). The DNPR is an ongoing administrative registry that contains records of all admissions and discharges from nonpsychiatric hospitals since 1977 and of all outpatient and emergency clinic visits since 1995 (Schmidt et al., 2015). Each hospital discharge or outpatient clinic visit is recorded with one primary diagnosis and up to 19 secondary diagnoses, coded according to the *International Classification of Diseases* 8th revision (ICD‐8) from 1977 to 1993 and the 10th revision (ICD‐10) thereafter.

Although we searched for hospital‐based diagnostic codes specific to type 2 diabetes, it is possible that some type 1 diabetes patients were erroneously coded with type 2 diabetes. To reduce this possible source of exposure misclassification, we constructed a subcohort consisting of patients diagnosed with type 2 diabetes who previously had redeemed a prescription for a noninsulin glucose‐lowering drug (e.g., metformin). Metformin is the recommended first‐line treatment for type 2 diabetes in Denmark (Christensen et al., 2016), and thus, it is unlikely that this subcohort contained patients with type 1 diabetes. As prescription data were available only from 2004 and onward, this subcohort was restricted to patients first diagnosed from 2005 and onward. We identified redeemed prescriptions from the Danish National Health Service Prescription Data (Johannesdottir et al., 2012), which contains data on all drug prescriptions redeemed in community pharmacies since January 1, 2004. Available data for each prescription include the date of redemption, the Anatomical Therapeutic Chemical Classification System code, type, and quantity.

In a validation study against general practice records, the positive predictive value of a diabetes diagnosis in the DNPR was found to be > 90% (Carstensen et al., 2011). Similarly, when measured against computerized height and weight measurements from hospital contacts, the positive predictive value of an obesity diagnosis in the DNPR was found to be 88% (Gribsholt et al., 2019).

### 2.3 General population comparison cohorts

To compare the risk of ALS after a type 2 diabetes or obesity diagnosis with that in the general population, we used the Civil Registration System (Schmidt et al., 2014) to construct three general population comparison cohorts (one for each patient cohort). For each type 2 diabetes and each obesity patient, we matched, with replacement, up to three persons from the general population on birth year and sex, requiring comparators to be alive on the index date of the matched patient (Heide‐Jørgensen et al., 2018). Comparators were then assigned an index date corresponding to that of their matched patient. The assignment of identical index dates in patients and general population comparators implies matching on calendar year. We required comparators to have no history of ALS or other motor neuron diseases, diabetes, or obesity before the index date.

### 2.4 Amyotrophic lateral sclerosis

The primary outcome was a primary or secondary hospital‐based inpatient or outpatient clinic discharge diagnosis of ALS, as recorded in the DNPR with ICD‐8 code 348 or ICD‐10 code G12.2 (ALS and other motor neuron diseases). A validation study found the positive predictive value of this definition to be 78% (95% CI: 71−84) when measured against medical records and applying the El Escorial criteria (Kioumourtzoglou et al., 2015). However, when also including clinically suspect ALS cases, the positive predictive value was 93% (95% CI: 88−96). Although the positive predictive value of this definition is reasonably high, in a supplementary analysis we also considered a stricter definition of ALS (ICD‐8 code 348.09, ICD‐10 code G12.2G) that excluded related forms of motor neuron disease.

### 2.5 Covariates

Smoking is associated with an increased risk of diabetes (Haire‐Joshu et al., 1999) and may also increase ALS risk (de Jong et al., 2012). We have previously shown that statins (primary and secondary therapeutic for patients with arterial cardiovascular disease) may increase ALS risk, especially in women (Skajaa et al., 2021). Thus, based on hospital‐based discharge diagnoses registered before the index date in the DNPR, we obtained information on chronic obstructive pulmonary disease (as an indicator of sustained smoking), myocardial infarction, stroke, hypercholesteremia, hypertension, atrial fibrillation, heart failure, cancer, and chronic kidney disease.

### 2.6 Statistical analyses

As ALS has a preclinical diagnostic phase, which often leads to a diagnostic delay (Eisen et al., 2014), we excluded the first year following the type 2 diabetes or obesity diagnosis to mitigate the potential identification of prevalent ALS diagnoses and to reduce the potential risk of surveillance bias associated with contact with the healthcare system. Thus, follow‐up started 1 year from the date of diagnosis until the first occurrence of a hospital‐based ALS diagnosis, death, emigration, or administrative study end (December 31, 2016). If a person from a comparison cohort was diagnosed with diabetes or obesity during follow‐up, he or she was transferred to the respective patient cohort and censored from the comparison cohort. For the type 2 diabetes analysis, we performed all analyses for both the overall cohort and the subcohort (2005−2016) of patients on glucose‐lowering drugs. We calculated the median follow‐up time, the number of ALS events, and incidence rates per 10,000 person‐years. We plotted the cumulative incidence of ALS, treating death as a competing risk (Andersen et al., 2012). We then used stratified Cox proportional hazards regression to calculate unadjusted and adjusted hazard ratios (HRs) of ALS, comparing the type 2 diabetes cohort with general population comparators and the obesity cohort with general population comparators. Time since the index date was the underlying time‐scale. In both unadjusted and adjusted models, the matching factors (birth year, sex, and calendar year) were controlled for by design, and not included in the models. The adjusted models additionally included the covariates described above.

### 2.7 Additional analyses

We examined effect modification on the relative scale by stratifying the analyses according to sex, age groups (≤50 years, 51−59 years, ≥60 years), number of comorbidities (0, 1, 2+), defined as any of the covariates, and calendar period of diagnosis (1980−1993, 1994−2016). When stratifying by sex, age, and calendar period, stratified Cox analyses were used; otherwise, ordinary Cox analyses were used, in which the matching was dissolved and the matching factors instead included in the model.

### 2.8 Sensitivity analyses

We performed a number of sensitivity analyses to examine the robustness of our results. First, we repeated the analyses using a stricter definition of ALS, that is, using codes specifically relating to ALS and not to other motor neuron diseases. Second, for patients diagnosed with type 2 diabetes or obesity during 2005−2016 (the period prescription data were available), we additionally adjusted for the use of statins and antihypertensives.

All statistical analyses were performed using SAS version 9.4 (SAS Institute, Cary, NC). The study was approved by the Danish Data Protection Agency (record no. 1‐16‐02‐1‐08). Table S1 lists all codes used in the study.

## 3 RESULTS

The analyses comprised 295,653 patients with type 2 diabetes diagnosed 1980−2016, 80,583 patients with type 2 diabetes diagnosed 2005−2016 on noninsulin glucose‐lowering drugs, and 312,108 patients with obesity diagnosed 1980−2016, as well as 977,110, 234,387, and 940,900 corresponding sex, age, and calendar year‐matched comparators from the general population (Tables 1 and 2). Of the 58,263 patients (20% of type 2 diabetes patients and 19% of obesity patients) with both a diagnosis of type 2 diabetes and obesity, 19,871 (34%) were diagnosed with type 2 diabetes followed by obesity, 21,309 (37%) were diagnosed with obesity followed by type 2 diabetes, and 17,083 (29%) were diagnosed with both diseases on the same day. In the type 2 diabetes cohorts, slightly more than half (55−56%) were men, the median age was 65 years, and around two‐thirds were diagnosed during 1994−2016 (the subcohort of patients on glucose‐lowering drugs were all diagnosed during this period). In the obesity cohort, women comprised around two‐thirds of the cohort, the median age was 42 years, and the vast majority (89%) were diagnosed during 1994−2016. Compared with general population comparators, both type 2 diabetes and obesity patients had higher prevalences of baseline comorbidities.

**TABLE 1:** Characteristics (*N*, %, unless stated otherwise) of patients with type 2 diabetes overall (1980−2016), patients with type 2 diabetes on glucose‐lowering drugs (2005−2016), and matched general population comparators.

	Type 2 diabetes overall cohort, *N* = 295,653	General population comparison cohort, *N* = 977,110	Type 2 diabetes subcohort, *N* = 80583	General population comparison cohort, *N* = 234,387
**Men**	161,288 (55%)	531,725 (54%)	45,332 (56%)	131,663 (56%)
**Median age, years (IQR)**	65 (54−74)	66 (55−75)	65 (54−74)	66 (55−73)
**Age groups**				
≤50 years	55,456 (19%)	168,679 (17%)	12,225 (15%)	36,482 (16%)
51−59 years	58,243 (20%)	181,242 (19%)	16,405 (20%)	48,675 (21%)
≥60	181,954 (62%)	627,189 (64%)	51,953 (65%)	149,230 (64%)
**Calendar period of diagnosis**				
1980−1993	74,419 (25%)	260,941 (27%)	–	–
1994−2016	221,234 (75%)	716,169 (73%)	80,583 (100%)	234,387 (100%)
**Comorbidities**				
Chronic obstructive pulmonary disease	22,566 (8%)	43,748 (5%)	7847 (10%)	14,308 (6%)
Myocardial infarction	18,069 (6%)	32,751 (3%)	5555 (7%)	8821 (4%)
Stroke	12,592 (4%)	27,681 (3%)	4688 (6%)	9928 (4%)
Hypercholesteremia	11,375 (4%)	20,209 (2%)	5997 (7%)	10,574 (5%)
Hypertension	43,240 (15%)	72,707 (7%)	18,446 (23%)	30,879 (13%)
Atrial fibrillation/flutter	17,961 (6%)	31,877 (3%)	6558 (8%)	11,290 (5%)
Heart failure	16,649 (6%)	21,355 (2%)	4620 (6%)	5883 (3%)
Cancer	21,742 (7%)	65,615 (7%)	8302 (10%)	21,992 (9%)
Chronic kidney disease	4027 (1%)	5761 (1%)	1080 (1%)	1986 (1%)

Abbreviation: IQR, interquartile range.

**TABLE 2:** Characteristics (*N*, %, unless stated otherwise) of patients with obesity (1980−2016) and matched general population comparators.

	Obesity cohort, *N* = 312,108	General population comparison cohort, *N* = 940,900
**Men**	79,896 (26%)	245,113 (26%)
**Median age, years (IQR)**	42 (30−59)	42 (30−59)
**Age groups**		
≤50 years	194,850 (62%)	578,011 (61%)
51−59 years	45,444 (15%)	137,664 (15%)
≥60	71,814 (23%)	225,225 (24%)
**Calendar period of diagnosis**		
1980−1993	35,515 (11%)	112,423 (12%)
1994−2016	276,593 (89%)	828,477 (88%)
**Comorbidities**		
Chronic obstructive pulmonary disease	21,753 (7%)	36,173 (4%)
Myocardial infarction	7893 (3%)	10,504 (1%)
Stroke	5747 (2%)	12,087 (1%)
Hypercholesteremia	8636 (3%)	11,289 (1%)
Hypertension	29,496 (10%)	38,052 (4%)
Atrial fibrillation/flutter	7182 (2%)	11,347 (1%)
Heart failure	6560 (2%)	6902 (1%)
Cancer	12,181 (4%)	33,120 (4%)
Chronic kidney disease	3133 (1%)	4186 (0.4%)

Abbreviation: IQR, interquartile range.

### 3.1 Type 2 diabetes versus general population

**FIGURE 1:**
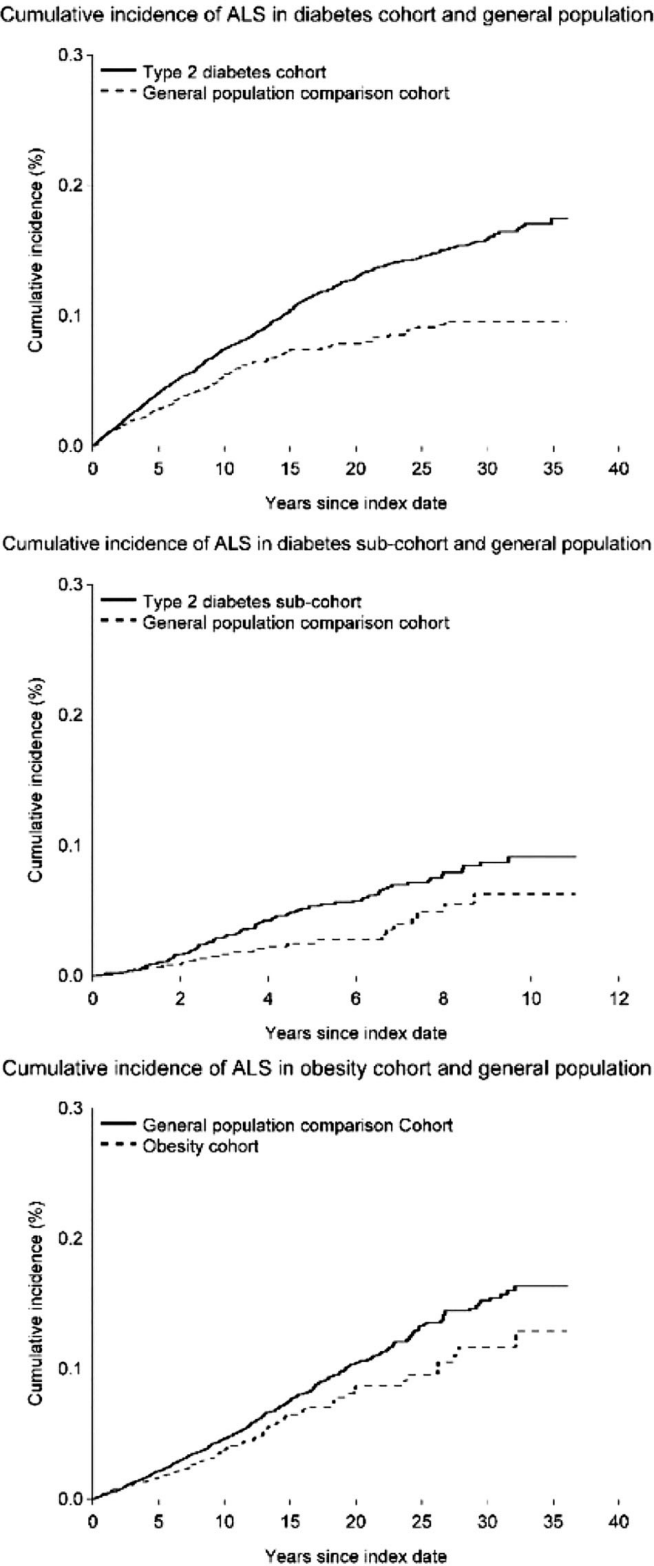
Events, incidence rates, and adjusted hazard ratios, comparing type 2 diabetes patients with matched general population comparators, overall and in subgroups according to sex, age groups, calendar period, and number of comorbidities. T2D: type‐2 diabetes; GP: general population; PY: person‐years; HR: hazard ratio. Adjusted HRs were for controlled for matching factors (sex, age, calendar year) and additionally adjusted for chronic obstructive pulmonary disease, myocardial infarction, stroke, hypercholesteremia, hypertension, atrial fibrillation, heart failure, cancer, chronic kidney disease. Comorbidities include chronic obstructive pulmonary disease, myocardial infarction, stroke, hypercholesteremia, hypertension, atrial fibrillation, heart failure, cancer, chronic kidney disease, and obesity.

**FIGURE 2:**
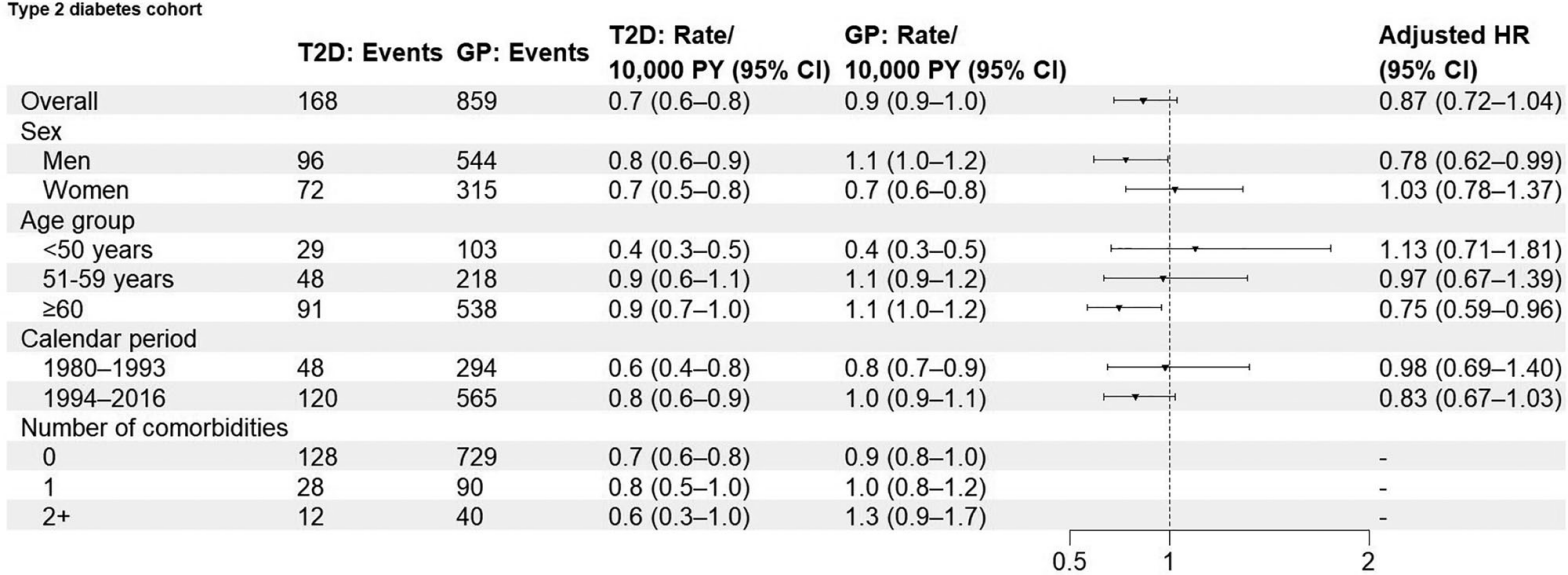
Events, incidence rates, and adjusted hazard ratios, comparing type 2 diabetes patients with a previously redeemed glucose‐lowering drug with matched general population comparators, overall and in subgroups according to sex, age groups, calendar period, and number of comorbidities. T2D: type‐2 diabetes; GP: general population; PY: person‐years; HR: hazard ratio. Adjusted HRs were for controlled for matching factors (sex, age, calendar year) and additionally adjusted for chronic obstructive pulmonary disease, myocardial infarction, stroke, hypercholesteremia, hypertension, atrial fibrillation, heart failure, cancer, chronic kidney disease. Comorbidities include chronic obstructive pulmonary disease, myocardial infarction, stroke, hypercholesteremia, hypertension, atrial fibrillation, heart failure, cancer, chronic kidney disease, and obesity.

**FIGURE 3:**
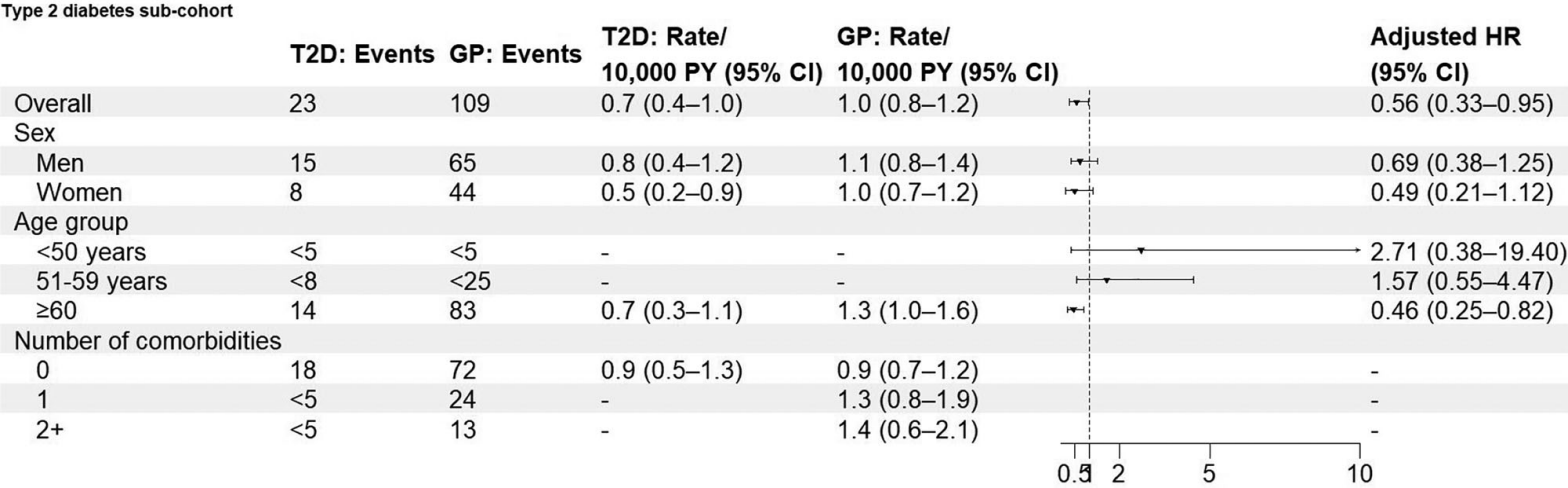
Cumulative incidence of amyotrophic lateral sclerosis among patients with type 2 diabetes, patients with type 2 diabetes with a noninsulin glucose‐lowering drug (subcohort), patients with obesity, and matched general population comparators.

In the overall cohort of patients with type 2 diabetes, we observed 168 incident cases of ALS (0.7 [95% CI: 0.6−0.8] per 10,000 person‐years) among patients and 859 ALS incident cases (0.9 [95% CI: 0.9−1.0] per 10,000 person‐years) among comparators (Figure 1). The median follow‐up was 6.1 years and 7.7 years, respectively. In the subcohort of patients with type 2 diabetes on noninsulin glucose‐lowering drugs, we observed 23 ALS events (0.7 [95% CI: 0.4−1.0] per 10,000 person‐years) among patients and 109 ALS events (1.0 [95% CI: 0.8−1.2] per 10,000 person‐years) among comparators, and the median follow‐up was 3.7 years and 4.0 years, respectively (Figure 2). Figure 3 depicts the cumulative incidence, considering death as a competing risk. The unadjusted and adjusted HRs were 0.86 (95% CI: 0.72−1.03) and 0.87 (95% CI: 0.72−1.04) in the overall cohort and 0.66 (95% CI: 0.41−1.04) and 0.56 (95% CI: 0.33−0.95) in the subcohort.

Examining effect measure modification on a relative scale, the association was present in men (adjusted HR: 0.78 [95% CI: 0.62−0.99]) and not in women (adjusted HR: 1.03 [95% CI: 0.78−1.37]) in the overall cohort. In the subcohort, the adjusted HR was 0.69 (95% CI: 0.38−1.25) in men and 0.49 (95% CI: 0.21−1.12) in women. The protective effect was present only in those aged ≥60 years in both the overall (adjusted HR: 0.75 [95% CI: 0.59−0.96]) and the subcohort (adjusted HR: 0.46 [95% CI: 0.25−0.82]). As well, the adjusted HR appeared to decrease with calendar period and an increasing number of comorbidities.

When using a stricter ALS definition, the adjusted HR was 0.83 (95% CI: 0.64−1.08) in the overall cohort and 0.66 (95% CI: 0.36−1.21) in the subcohort. When additionally adjusting for statins and antihypertensives, among patients diagnosed during 2005−2016, the adjusted HR was 0.69 (95% CI: 0.48−1.00) in the overall cohort and 0.56 (95% CI: 0.33−0.95) in the subcohort.

### 3.2 Obesity versus general population

**FIGURE 4:**
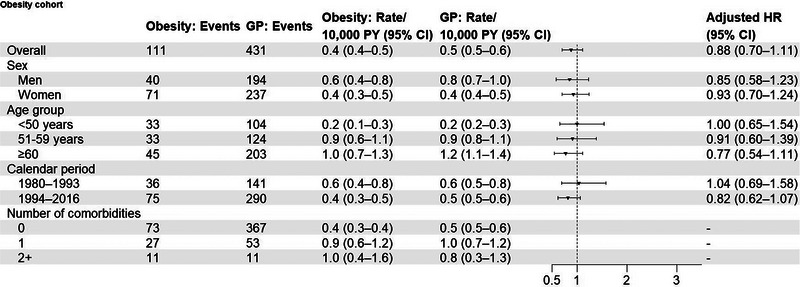
Events, incidence rates, and adjusted hazard ratios, comparing obesity patients with matched general population comparators in subgroups according to sex, age groups, calendar period, and number of comorbidities. GP: general population; PY: person‐years; HR: hazard ratio. Adjusted HRs were for controlled for matching factors (sex, age, calendar year) and additionally adjusted for chronic obstructive pulmonary disease, myocardial infarction, stroke, hypercholesteremia, hypertension, atrial fibrillation, heart failure, cancer, chronic kidney disease. Comorbidities include chronic obstructive pulmonary disease, myocardial infarction, stroke, hypercholesteremia, hypertension, atrial fibrillation, heart failure, cancer, chronic kidney disease, and obesity.

During a median follow‐up of 6.4 years among obesity patients and 6.5 years among comparators, we observed 111 ALS events (0.4 [95% CI: 0.4−0.5] per 10,000 person‐years) among obesity patients and 431 ALS events (0.5 [95% CI: 0.5−0.6] per 10,000 person‐years) among comparators (Figure 4). Figure 3 shows the cumulative incidence over time. The unadjusted and adjusted HRs were 0.89 (95% CI: 0.71−1.10) and 0.88 (95% CI: 0.70−1.11).

Although estimates were fairly imprecise, we observed a similar pattern of effect measure modification for obesity as for type 2 diabetes: Adjusted HRs were lower in men than women, in those aged ≥60 years than those younger, and in patients diagnosed during 1994–2016. However, the HR appeared to increase with an increasing number of comorbidities, in contrast to that observed for type 2 diabetes.

Using the stricter ALS definition, the adjusted HR was 0.82 (95% CI: 0.60−1.13). When additionally adjusting for statins and antihypertensives, the adjusted HR was 1.04 (95% CI: 0.69−1.55).

## 4 DISCUSSION

In this large population‐based cohort study with more than 30 years of observation time, an inpatient or outpatient hospital‐based diagnosis of type 2 diabetes was associated with a reduced rate of ALS compared with matched general population comparators, specifically among patients aged 60 years or above and more clearly in men than women. A hospital‐based diagnosis of obesity was also associated with a marginally reduced rate of ALS, but the effect estimate was less precise. Despite the observed protective effects, the absolute rate differences were small.

Some potential limitations must be discussed. First, ALS has an insidious onset, which may have led to the inclusion of diabetes and obesity patients with prevalent ALS. However, we mitigated this potential risk, as well as the potential risk of surveillance bias arising from contact with the healthcare system associated with the diagnostic workup of diabetes or obesity, by excluding from the analysis the first year following diagnosis. Second, we cannot rule out misclassification of both the exposure (i.e., type 2 diabetes or obesity) and outcome. While the completeness of the obesity diagnosis is low in the DNPR, the positive predictive value of both diabetes and obesity diagnoses is high (88−90%) (Carstensen et al., 2011; Gribsholt et al., 2019). Regardless, any potential exposure misclassification would likely bias the associations toward unity, that is, masking a true protective effect. The analysis of the subcohort of type 2 diabetes patients on noninsulin glucose‐lowering drugs, undertaken to reduce misclassification with type 1 diabetes, showed a lower hazard, further demonstrating that the overall results may have been conservative. On the other hand, the association decreased with increasing calendar period, and patients comprising the subcohort were diagnosed in more recent years; thus, the more pronounced findings for the subcohort could also be explained by calendar time. Our patient inclusion was restricted to those with an inpatient or outpatient hospital‐based diagnosis, thereby not capturing patients with mild disease. However, we probably identified some of these patients in the subcohort of type 2 diabetes patients on glucose‐lowering drugs, because prescription data are available for the entire Danish population. Third, as our study was observational, we cannot rule out residual or unknown confounding. Of note, we probably only partly adjusted for the confounding effect of smoking, using a hospital‐based diagnosis of chronic obstructive pulmonary disease as a proxy for sustained smoking. We also lacked data on physical activity. However, the small differences between unadjusted and adjusted HRs leave little indication that residual confounding could explain the results.

Our findings agree with most previous literature indicating that cardiometabolic conditions, including type 2 diabetes and obesity, are associated with a reduced risk of ALS risk (D'Ovidio et al., 2018; Kioumourtzoglou et al., 2015; Mariosa et al., 2015; O'Reilly et al., 2013; Tsai et al., 2019). The effect sizes observed in this study regarding both associations are at par with those reported earlier. However, unlike previous reports, we found an indication of a possible sex difference, as the protective association of type 2 diabetes in the overall cohort was present in men and not in women. We are unaware of any explanation for this finding. Nevertheless, we have previously found a large sex‐differential effect when examining the effect of statins on ALS risk, as the observed adverse effect of statins was present only in women (Skajaa et al., 2021). In alignment with existing knowledge (Kioumourtzoglou et al., 2015; Mariosa et al., 2015; Tsai et al., 2019), we observed clear effect modification by age, as any type 2 diabetes benefit was present only in those aged 60 years or older. Kioumourtzoglou et al. (2015) hypothesized that the null or harmful association found for younger ages could be explained by differences between type 1 and 2 diabetes, that is, a type 1 diabetes diagnosis is most often given at younger ages. As noted above, we cannot rule out misclassification between type 1 and 2 diabetes despite only including diagnostic codes specific for type 2 diabetes. However, although estimates were much less precise, the same age pattern was observed for obesity, which may warrant another explanation.

The mechanisms explaining the theoretical benefits of cardiometabolic conditions, including type 2 diabetes and obesity, are not well‐understood. ALS appears to be a disease not restricted to the central nervous system but may have more widespread effects, including effects on energy metabolism (Dupuis et al., 2011). In most ALS patients, energy uptake is reduced while energy expenditure is increased, leading to reduced fat depots (Vandoorne et al., 2018). This may explain the protective effects observed for obesity; however, assuming causality, the direction of the cause‐effect association is convoluted as the ALS onset is insidious, and many patients may simply be pre‐symptomatic for ALS, and thus undiagnosed, at the time of obesity diagnosis. On the other hand, such an effect could also be explained by elevated lipid and cholesterol levels (Dupuis et al., 2008; Sutedja et al., 2011). Regarding diabetes, most evidence suggests that a potential protective effect on ALS risk is restricted to type 2 diabetes, a condition associated with insulin resistance (Kioumourtzoglou et al., 2015; Mariosa et al., 2015). It is possible that metformin treatment, the first‐line treatment of type 2 diabetes in Denmark (Christensen et al., 2016), could exert some benefit. While animal studies have yielded mixed results (Kaneb et al., 2011; Sun et al., 2013), a Swedish case‐control study suggested that patients using antidiabetic drugs had a 35% to a 10% lower risk of ALS compared with matched nonusers (Mariosa et al., 2020).

In summary, our cohort study found protective effects associated with hospital‐based diagnoses of type 2 diabetes and obesity on ALS risk. The effects were most clearly observed among men and those aged 60 years or older. Despite these protective effects, the absolute rate differences were small, preventing clear clinical implications of these findings. Findings add to existing knowledge that energy metabolism plays a role in ALS occurrence.

## AUTHOR CONTRIBUTIONS

All authors contributed to the design of the study. EHP and HTS acquired the data. All authors directed the analyses, which was carried out by SKS. NS and EBR wrote the initial draft. All authors contributed to the discussion and interpretation of the results, which secured the intellectual content of the manuscript. All authors accepted the final version for submission.

## FUNDING

No targeted funding for this study.

## CONFLICT OF INTEREST STATEMENT

The Department of Clinical Epidemiology, Aarhus University Hospital, receives funding for other studies from companies in the form of research grants to (and administered by) Aarhus University. None of these studies has any relation to the present study.

### PEER REVIEW

The peer review history for this article is available at https://publons.com/publon/10.1002/brb3.3007.

## Supporting information

Supplemental Table S1. International Classification of Diseases (ICD) and Anatomical Therapeutic Chemical Classification System (ATC) codes used in the study.

## Data Availability

The data that support the findings of this study are available from Statistics Denmark.

## References

[brb33007-bib-0001] Andersen, P. K. , Geskus, R. B. , de Witte, T. , & Putter, H. (2012). Competing risks in epidemiology: Possibilities and pitfalls. International Journal of Epidemiology, 41(3), 861–870. 10.1093/ije/dyr213 22253319 PMC3396320

[brb33007-bib-0002] Brown, R. H. , & Al‐Chalabi, A. (2017). Amyotrophic lateral sclerosis. New England Journal of Medicine, 377(2), 162–172. 10.1056/NEJMra1603471 28700839

[brb33007-bib-0003] Carstensen, B. , Kristensen, J. K. , Marcussen, M. M. , & Borch‐Johnsen, K. (2011). The national diabetes register. Scandinavian Journal of Public Health, 39(7), Suppl 58–61. 10.1177/1403494811404278 21775353

[brb33007-bib-0004] Christensen, D. H. , Rungby, J. , & Thomsen, R. W. (2016). Nationwide trends in glucose‐lowering drug use, Denmark, 1999–2014. Clinical Epidemiology, 8, 381–387. 10.2147/CLEP.S113211 27789974 PMC5072551

[brb33007-bib-0005] de Jong, S. W. , Huisman, M. H. B. , Sutedja, N. A. , van der Kooi, A. J. , de Visser, M. , Schelhaas, H. J. , Fischer, K. , Veldink, J. H. , & van den Berg, L. H. (2012). Smoking, alcohol consumption, and the risk of amyotrophic lateral sclerosis: A population‐based study. American Journal of Epidemiology, 176(3), 233–239. 10.1093/aje/kws015 22791740

[brb33007-bib-0006] Del Aguila, M. A. , Longstreth, W. T. , McGuire, V. , Koepsell, T. D. , & van Belle, G. (2003). Prognosis in amyotrophic lateral sclerosis: A population‐based study. Neurology, 60(5), 813–819. 10.1212/01.wnl.0000049472.47709.3b 12629239

[brb33007-bib-0007] D'Ovidio, F. , d’ Errico, A. , Carnà, P. , Calvo, A. , Costa, G. , & Chiò, A. (2018). The role of pre‐morbid diabetes on developing amyotrophic lateral sclerosis. European Journal of Neurology, 25(1), 164–170. 10.1111/ene.13465 28921834

[brb33007-bib-0008] Dupuis, L. , Corcia, P. , Fergani, A. , Gonzalez De Aguilar, J. L. , Bonnefont‐Rousselot, D. , Bittar, R. , Seilhean, D. , Hauw, J. J. , Lacomblez, L. , Loeffler, J. P. , & Meininger, V. (2008). Dyslipidemia is a protective factor in amyotrophic lateral sclerosis. Neurology, 70(13), 1004–1009. 10.1212/01.wnl.0000285080.70324.27 18199832

[brb33007-bib-0009] Dupuis, L. , Pradat, P.‐F. , Ludolph, A. C. , & Loeffler, J.‐P. (2011). Energy metabolism in amyotrophic lateral sclerosis. Lancet Neurology, 10(1), 75–82. 10.1016/S1474-4422(10)70224-6 21035400

[brb33007-bib-0010] Eisen, A. , Kiernan, M. , Mitsumoto, H. , & Swash, M. (2014). Amyotrophic lateral sclerosis: A long preclinical period? Journal of Neurology, Neurosurgery, and Psychiatry, 85(11), 1232–1238. 10.1136/jnnp-2013-307135 24648037

[brb33007-bib-0011] Gribsholt, S. B. , Pedersen, L. , Richelsen, B. , & Thomsen, R. W. (2019). Validity of ICD‐10 diagnoses of overweight and obesity in Danish hospitals. Clinical Epidemiology, 11, 845–854. 10.2147/CLEP.S214909 31572015 PMC6748036

[brb33007-bib-0012] Haire‐Joshu, D. , Glasgow, R. E. , & Tibbs, T. L. (1999). Smoking and diabetes. Diabetes Care, 22(11), 1887–1898. 10.2337/diacare.22.11.1887 10546025

[brb33007-bib-0013] Heide‐Jørgensen, U. , Adelborg, K. , Kahlert, J. , Sørensen, H. T. , & Pedersen, L. (2018). Sampling strategies for selecting general population comparison cohorts. Clinical Epidemiology, 10, 1325–1337. 10.2147/CLEP.S164456 30310326 PMC6165733

[brb33007-bib-0014] Hollinger, S. K. , Okosun, I. S. , & Mitchell, C. S. (2016). Antecedent disease and amyotrophic lateral sclerosis: What is protecting whom? Frontier Neurology, 7, 47. 10.3389/fneur.2016.00047 PMC4810157 27065942

[brb33007-bib-0015] Ingre, C. , Roos, P. M. , Piehl, F. , Kamel, F. , & Fang, F. (2015). Risk factors for amyotrophic lateral sclerosis. Clinical Epidemiology, 7, 181–193. 10.2147/CLEP.S37505 25709501 PMC4334292

[brb33007-bib-0016] Johannesdottir, S. A. , Horváth‐Puhó, E. , Ehrenstein, V. , Schmidt, M. , Pedersen, L. , & Sørensen, H. T. (2012). Existing data sources for clinical epidemiology: The danish national database of reimbursed prescriptions. Clinical Epidemiology, 4, 303–313. 10.2147/CLEP.S37587 23204870 PMC3508607

[brb33007-bib-0017] Kaneb, H. M. , Sharp, P. S. , Rahmani‐Kondori, N. , & Wells, D. J. (2011). Metformin treatment has no beneficial effect in a dose‐response survival study in the SOD1(G93A) mouse model of ALS and is harmful in female mice. PLoS ONE, 6(9), e24189. 10.1371/journal.pone.0024189 21909419 PMC3164704

[brb33007-bib-0018] Kioumourtzoglou, M.‐A. , Rotem, R. S. , Seals, R. M. , Gredal, O. , Hansen, J. , & Weisskopf, M. G. (2015). Diabetes mellitus, obesity, and diagnosis of amyotrophic lateral sclerosis: A population‐based study. JAMA Neurology, 72(8), 905–911. 10.1001/jamaneurol.2015.0910 26030836 PMC4975611

[brb33007-bib-0019] Kioumourtzoglou, M.‐A. , Seals, R. M. , Himmerslev, L. , Gredal, O. , Hansen, J. , & Weisskopf, M. G. (2015). Comparison of diagnoses of amyotrophic lateral sclerosis by use of death certificates and hospital discharge data in the Danish population. Amyotrophic Lateral Sclerosis and Frontotemporal Degeneration, 16(3–4), 224–229. 10.3109/21678421.2014.988161 25946516 PMC4675622

[brb33007-bib-0020] Lekoubou, A. , Matsha, T. E. , Sobngwi, E. , & Kengne, A. P. (2014). Effects of diabetes mellitus on amyotrophic lateral sclerosis: A systematic review. BMC Research Notes, 7, 171. 10.1186/1756-0500-7-171 24661645 PMC3987838

[brb33007-bib-0021] Mariosa, D. , Kamel, F. , Bellocco, R. , Ronnevi, L. ‐ O. , Almqvist, C. , Larsson, H. , Ye, W. , & Fang, F. (2020). Antidiabetics, statins and the risk of amyotrophic lateral sclerosis. European Journal of Neurology, 27(6), 1010–1016. 10.1111/ene.14190 32097525 PMC10957794

[brb33007-bib-0022] Mariosa, D. , Kamel, F. , Bellocco, R. , Ye, W. , & Fang, F. (2015). Association between diabetes and amyotrophic lateral sclerosis in Sweden. European Journal of Neurology, 22(11), 1436–1442. 10.1111/ene.12632 25600257 PMC4506907

[brb33007-bib-0023] Mitchell, C. S. , Hollinger, S. K. , Goswami, S. D. , Polak, M. A. , Lee, R. H. , & Glass, J. D. (2015). Antecedent disease is less prevalent in amyotrophic lateral sclerosis. Neurodegenerative Diseases, 15(2), 109–113. 10.1159/000369812 25720304 PMC4417009

[brb33007-bib-0024] O'Reilly, É. J. , Wang, H. , Weisskopf, M. G. , Fitzgerald, K. C. , Falcone, G. , McCullough, M. L. , Thun, M. , Park, Y. , Kolonel, L. N. , & Ascherio, A. (2013). Premorbid body mass index and risk of amyotrophic lateral sclerosis. Amyotrophic Lateral Sclerosis and Frontotemporal Degeneration, 14(3), 205–211. 10.3109/21678421.2012.735240 23134505 PMC3615420

[brb33007-bib-0025] Schmidt, M. , Pedersen, L. , & Sørensen, H. T. (2014). The Danish Civil Registration System as a tool in epidemiology. European Journal of Epidemiology, 29(8), 541–549. https://doi.org.ez.statsbiblioteket.dk:2048/10.1007/s10654‐014‐9930‐3 24965263 10.1007/s10654-014-9930-3

[brb33007-bib-0026] Schmidt, M. , Schmidt, S. A. J. , Adelborg, K. , Sundbøll, J. , Laugesen, K. , Ehrenstein, V. , & Sørensen, H. T. (2019). The Danish health care system and epidemiological research: From health care contacts to database records. Clinical Epidemiology, 11, 563–591. 10.2147/CLEP.S179083 31372058 PMC6634267

[brb33007-bib-0027] Schmidt, M. , Schmidt, S. A. J. , Sandegaard, J. L. , Ehrenstein, V. , Pedersen, L. , & Sørensen, H. T. (2015). The Danish National Patient Registry: A review of content, data quality, and research potential. Clinical Epidemiology, Published online November 2015, 449. 10.2147/CLEP.S91125 26604824 PMC4655913

[brb33007-bib-0028] Seelen, M. , van Doormaal, P. T. C. , Visser, A. E. , Huisman, M. H. , Roozekrans, M. H. , de Jong, S. W. , van der Kooi, A. J. , de Visser, M. , Voermans, N. C. , Veldink, J. H. , & van den Berg, L. H. (2014). Prior medical conditions and the risk of amyotrophic lateral sclerosis. Journal of Neurology, 261(10), 1949–1956. 10.1007/s00415-014-7445-1 25059395

[brb33007-bib-0029] Skajaa, N. , Bakos, I. , Horváth‐Puhó, E. , Henderson, V. W. , Lash, T. L. , & Sørensen, H. T. (2021). Statin initiation and risk of amyotrophic lateral sclerosis: A Danish population‐based cohort study. Epidemiology Cambridge, Mass, 32(5), 756–762. 10.1097/EDE.0000000000001384 34183532

[brb33007-bib-0030] Sun, H. , Knippenberg, S. , Thau, N. , Ragancokova, D. , Körner, S. , Huang, D. , Dengler, R. , Döhler, K. , & Petri, S. (2013). Therapeutic potential of N‐acetyl‐glucagon‐like peptide‐1 in primary motor neuron cultures derived from non‐transgenic and SOD1‐G93A ALS mice. Cellular and Molecular Neurobiology, 33(3), 347–357. 10.1007/s10571-012-9900-9 23271639 PMC11498007

[brb33007-bib-0031] Sun, Y. , Lu, C.‐J. , Chen, R.‐C. , Hou, W.‐H. , & Li, C.‐Y. (2015). Risk of amyotrophic lateral sclerosis in patients with diabetes: A nationwide population‐based cohort study. Journal of Epidemiology, 25(6), 445–451. 10.2188/jea.JE20140176 25947580 PMC4444499

[brb33007-bib-0032] Sutedja, N. A. , van der Schouw, Y. T. , Fischer, K. , Sizoo, E. M. , Huisman, M. H. B. , Veldink, J. H. , & Van den Berg, L. H. (2011). Beneficial vascular risk profile is associated with amyotrophic lateral sclerosis. Journal of Neurology, Neurosurgery, and Psychiatry, 82(6), 638–642. 10.1136/jnnp.2010.236752 21471184

[brb33007-bib-0033] Tsai, C.‐P. , Lee, J. K.‐W. , & Lee, C. T.‐C. (2019). Type II diabetes mellitus and the incidence of amyotrophic lateral sclerosis. Journal of Neurology, 266(9), 2233–2243. 10.1007/s00415-019-09405-x 31152300

[brb33007-bib-0034] VanderWeele, T. J. , & Ding, P. (2017). Sensitivity analysis in observational research: Introducing the E‐value. Annals of Internal Medicine, 167(4), 268–274. 10.7326/M16-2607 28693043

[brb33007-bib-0035] Vandoorne, T. , De Bock, K. , & Van Den Bosch, L. (2018). Energy metabolism in ALS: An underappreciated opportunity? Acta Neuropathologica, 135(4), 489–509. 10.1007/s00401-018-1835-x 29549424 PMC5978930

[brb33007-bib-0036] van Es, M. A. , Hardiman, O. , Chio, A. , Al‐Chalabi, A. , Pasterkamp, R. J. , Veldink, J. H. , & van den Berg, L. H. (2017). Amyotrophic lateral sclerosis. Lancet, London, England, 390(10107), 2084–2098. 10.1016/S0140-6736(17)31287-4 28552366

