# Supplementary material for: Type 2 diabetes, obesity, and risk of amyotrophic lateral sclerosis: A population‐based cohort study
Source: Brain Behav. 2023 Apr 19;13(6):e3007. doi: 10.1002/brb3.3007 (PMC10275529; doi:10.1002/brb3.3007)
Supplement: Supplementary file 1 — Supplemental Table S1. International Classification of Diseases (ICD) and Anatomical Therapeutic Chemical Classification System (ATC) codes used in the study. [file BRB3-13-e3007-s001.docx]

**SUPPLEMENTAL MATERIAL**

**Supplemental Table I.** *International Classification of Diseases* (ICD) and *Anatomical Therapeutic Chemical Classification System* (ATC) codes used in the study.

|  | **ICD-8** | **ICD-10** | **ATC** |
| --- | --- | --- | --- |
| **Exposure** |  |  |  |
| Type 2 diabetes | 250 | E11 |  |
| Obesity | 277 | E65-E68 |  |
| **Outcome** |  |  |  |
| Amyotrophic lateral sclerosis or other motor neuron diseases | 348 | G12.2 |  |
| Amyotrophic lateral sclerosis | 34809 | G12.2G |  |
| **Covariates** |  |  |  |
| Chronic obstructive pulmonary disease | 490-493, 515- 518 | J40-J47, J60-J67, J68.4, J70.1, J70.3, J84.1, J92.0, J96.1, J98.2-J98.3 |  |
| Myocardial infarction | 410 | I21 |  |
| Stroke | 430, 431, 433, 434 | I60, I61, I63, I64 |  |
| Hypercholesteremia | 27200 | E780 |  |
| Hypertension | 400-404 | I10-I15, I67.4 |  |
| Atrial fibrillation | 42793, 42794 | I48 |  |
| Heart failure | 42709, 42710, 42711, 42719, 42899, 78249 | I500, I501, I502, I503, I508, I509, I110, I130, I132, I420, I426, I427, I428, I429 |  |
| Cancer | 140–209 | C00–C99 |  |
| Chronic kidney disease | 249.02, 250.02, 753.10-753.19, 582, 583, 584, 590.09, 593.20, 792 | E10.2, E11.2, E14.2, N03, N05, N11.0, N14, N16, N18-N19, N26.9, Q61.1-Q61.4 |  |
| Statins |  |  | C10AA |
| Antihypertensives |  |  | ≥2 of the following classes within 180 days before index date: α adrenergic blockers (C02A, C02B, C02C), non-loop diuretics (C02DA, C02L, C03A, C03B, C03D, C03E, C03X, C07C, C07D, C08G, C09BA, C09DA, C09XA52), vasodilators (C02DB, C02DD, C02DG, C04, C05), beta blockers (C07), calcium channel blockers (C07F, C08, C09BB, C09DB), and renin-angiotensin system inhibitors (C09) |
| Insulin |  |  | A10A |
| Glucose-lowering drugs |  |  | A10B |
| Metformin |  |  | A10BA02 |
